# Phenotyping of *Salvia miltiorrhiza* Roots Reveals Associations between Root Traits and Bioactive Components

**DOI:** 10.34133/plantphenomics.0098

**Published:** 2023-10-02

**Authors:** Junfeng Chen, Yun Wang, Peng Di, Yulong Wu, Shi Qiu, Zongyou Lv, Yuqi Qiao, Yajing Li, Jingfu Tan, Weixu Chen, Ma Yu, Ping Wei, Ying Xiao, Wansheng Chen

**Affiliations:** ^1^ The SATCM Key Laboratory for New Resources & Quality Evaluation of Chinese Medicine, Institute of Chinese Materia Medica, Shanghai University of Traditional Chinese Medicine, Shanghai 201203, China.; ^2^ School of Medicine, Shanghai University, Shanghai 200444, China.; ^3^ State Local Joint Engineering Research Center of Ginseng Breeding and Application, Jilin Agricultural University, Changchun 130118, China.; ^4^ School of Computer Science, Sichuan Normal University, Chengdu 610066, China.; ^5^ Shangyao Huayu (Linyi) Traditional Chinese Resources Co., Ltd., Linyi 276000, China.; ^6^ School of Life Science and Engineering, Southwest University of Science and Technology, Mianyang 621010, Sichuan, China.; ^7^ Sichuan Academy of Traditional Chinese Medicine, Chengdu 610041, China.; ^8^ Department of Pharmacy, Changzheng Hospital, Second Military Medical University, Shanghai 200003, China.

## Abstract

Plant phenomics aims to perform high-throughput, rapid, and accurate measurement of plant traits, facilitating the identification of desirable traits and optimal genotypes for crop breeding. *Salvia miltiorrhiza* (Danshen) roots possess remarkable therapeutic effect on cardiovascular diseases, with huge market demands. Although great advances have been made in metabolic studies of the bioactive metabolites, investigation for *S*. *miltiorrhiza* roots on other physiological aspects is poor. Here, we developed a framework that utilizes image feature extraction software for in-depth phenotyping of *S*. *miltiorrhiza* roots. By employing multiple software programs, *S. miltiorrhiza* roots were described from 3 aspects: agronomic traits, anatomy traits, and root system architecture. Through *K*-means clustering based on the diameter ranges of each root branch, all roots were categorized into 3 groups, with primary root-associated key traits. As a proof of concept, we examined the phenotypic components in a series of randomly collected *S*. *miltiorrhiza* roots, demonstrating that the total surface of root was the best parameter for the biomass prediction with high linear regression correlation (*R*^2^ = 0.8312), which was sufficient for subsequently estimating the production of bioactive metabolites without content determination. This study provides an important approach for further grading of medicinal materials and breeding practices.

## Introduction

The advancement of image recognition technology and the progress of algorithms have led to the emergence of plant phenomics as a new discipline for comprehending plant phenotypes [1]. This discipline is poised to accelerate the research on plant physiology, genetics, and breeding [2]. Among various physiological aspects such as genome, transcriptome, and metabolism, plant phenotyping poses a significant bottleneck in achieving a comprehensive understanding of plant physiology. Traditional plant phenotyping heavily relies on manual observations and measurements, which are labor-intensive, tedious, time-consuming, and costly, and have lower throughput. Over the past decade, plant phenomics has made significant advancements facilitated by automated phenotypic systems, imaging techniques, and algorithm-based machine learning (ML) methods [3]. Plant phenomics refers to the nondestructive and accurate acquisition of high-dimensional phenotypic data on an organism-wide scale across plant developmental stages, from an individual plant to field scale [1]. Generally, plant phenotyping includes 2 major investigation aspects. The first is phenotypic data assessment, including high-throughput image acquisition, image processing, and feature extraction. Subsequently, diverse data mining strategies are derived to comprehensively investigate the collected phenotypic data for different purposes [4].

Based on a series of optical sensors, plant species, and study purposes, various phenotyping pipelines have been developed, which enable data acquisition at different scales from different levels, including cellular, organ, whole plant, and field [5]. Currently, plant phenomics primarily supports breeding research in major crops such as rice, wheat, and maize [6]. The focus lies in identifying desirable crop traits by analyzing phenotypic variations, throughout the growth and development stages, considering biotic and abiotic stress factors, as well as various nutritional conditions [4,7–9]. Combined with the high-throughput genomics technology and algorithms, plant phenomics can further assist in bridging the gaps between genotype and phenotype, which would help to hasten crop breeding.

Besides crops, medicinal plants represent another extensively cultivated plant category with significant therapeutic and economic values. Presently, investigations on medicinal plants are mainly focused on the active metabolites and their biosynthesis. To this end, integrated omics studies have been accomplished, in order to pave a way for standardized authentication of the plant materials and bioengineering of the metabolic pathways in medicinal plants [10]. Nevertheless, in comparison to other research aspects (i.e., genomics, transcriptomics, proteomics, and metabolomics), the progress of phenomics for medicinal plants is obviously insufficient, which may be related to the diversity of medicinal plant species. Moreover, owing to the accumulation of particular beneficial metabolites, the root plays a crucial role in obtaining the active ingredients in various medicinal plants. However, as the hidden half of the plant, phenotyping of the root system presents greater difficulties compared to that of the aerial part, making it a challenging subject in the field of plant phenomics [11]. For the estimation of biomass and agronomic parameters of roots, a high-throughput and high-resolution root scanning system, graphic processing, and data extraction are required [12]. Additionally, the characterization of root system architecture (RSA) is another important aspect of root phenotyping. RSA is a multifaceted concept shaped by parameters, density, and topology of the root system [13,14]. Therefore, a comprehensive perspective is necessary to fully characterize RSA.

*Salvia miltiorrhiza* Bge., belonging to the Lamiaceae family, is a commonly used Chinese herbal medicine in curing cardiovascular disease. The rhizome of *S*. *miltiorrhiza* is rich in diterpenoids and phenolic acids as main active components [15]. Certain bioactive metabolites as tanshinones (TAs) and lithospermic acid B (LAB) are abundantly accumulated in *S*. *miltiorrhiza* roots [16] with significant species specificities. Therefore, there is an increasing market demand for this herb, accompanied by a higher request for breeding research. Although several current studies that focused on the biosynthesis and regulation of active metabolites in *S. miltiorrhiza* have achieved a series of successful breeding in promoting these metabolites [17,18]. Limited studies have focused on the agronomic characters of *S. miltiorrhiza* [19], leading to a blank research field of this herb. However, if the genetic laws of specific phenotypes can be quantified and analyzed on the basis of rapid screening of key agronomic trait sets, the monitoring and regulation of *S. miltiorrhiza* or other herbs at different levels of cells, organs, and populations will inevitably improve breeding, cultivation, and agricultural field production.

Here, we established a workflow to capture the phenotypic traits of *S. miltiorrhiza* roots using multiple published root phenotyping software. Data mining of phenotypic traits was further carried out to classify the root samples and identify the key root features. As a proof of concept, we analyzed the correlation between root phenotypic traits and their accumulation of bioactive compounds, providing a prediction model for tanshinone content in roots without metabolic determination.

## Materials and Methods

### Plant materials and root sections

All test root samples were the cultivated variety of *S. miltiorrhiza* provided by Shangyao Huayu (Linyi) Traditional Chinese Resources Co., Ltd. Seedlings that had grown for about 10 months after sowing in the same nursery plot were selected and planted in March in a trial field (located in 117°63′E, 35°50′N, Pingyi County, Shandong Province, China) in a natural environment without human intervention. Randomly selected *S. miltiorrhiza* plants were numbered, while fresh roots were harvested intact during the harvesting period in October of the same year as the test material. The underground roots were completely excavated after the aerial part was eliminated. As a medicinal material, the main roots and the lateral roots with a diameter greater than a certain range will be chosen for further production of decoction pieces. Therefore, all absorbing roots were removed in this study. All roots were washed to remove soil for further image capture. After whole-root imaging, paraffin cross-sections from the primary root of each sample were produced according to a previous method [20], and used for further imaging and metabolic analysis.

### Image capture and feature extraction

The roots were removed from the soil and cleaned and passed through a 1-mm sieve. After removing the adventitious roots, the roots were arranged in a Perspex tray with a shallow water film to avoid overlapping of the roots. For agronomic traits extraction, a high-resolution scanner equipped with the WinRHIZO root scanning system was applied for whole root scanning in both projection and reflection modes. These images were in the 2D WinRhizo images (size 300 dpi, 1,200 × 1,700 pixels). This procedure required ∼1 min for each destructively harvested sample. The obtained images were analyzed using WinRHIZO and RhizoVision explorer [21,22], respectively. The data generated by the 2 software are highly consistent [22], but a preliminary analysis indicated that the WinRhizo software did not reliably measure the number of branches, whereas RhizoVision can provide more refined root ranging. Therefore, parameters produced by RhizoVision were mainly chosen for further analysis. To reduce false identification, parameters obtained from projection images were used for further investigations. To reduce the impact of root cross superposition, some large root samples were disassembled into simple roots for image acquisition and analysis. Diameter ranging was performed by RhizoVision. The ranges (in millimeters) were 0 to 20.00, 20.00 to 40.00, 40.00 to 60.00, and above 60.00.

Paraffin cross-sections from the primary root of each sample were used to obtain the anatomy traits of *S. miltiorrhiza* root. The sections were scanned by a slide scanner system (Pannoramic 250 FLASH, Sysmex, Germany) and then analyzed using RootScan to obtain information on tissue distributions [23] (Fig. S2). The cell layers of cortex, stele, and xylem tissues were distinguished semi-manually, and their area and proportion were estimated.

The landmark method RootScape was used to characterize the skeleton of root system [24]. A 9-landmark set was designed to mainly describe the occupation of primary root and first-order lateral roots. They were the start and end of the primary root, the first lateral root on the main root, and the widest points of the whole root system (Fig. 3A). A matrix was then generated including the position information of each landmark for each sample. The distance between each landmark pair was calculated by the following formula: sqrt((x2 − x1)^2^ + (y2 − y1)^2^), according to which, principal component analysis (PCA) and hierarchical clustering methods were performed to cluster the root samples.

### Statistical analysis

Statistical analysis and graphic generation were performed by linear regression, *K*-means clustering, and normal distribution in R language. Several ML methods were performed to evaluate the accuracy of classification using unsupervised models according to algorithms reported by Xu et al. [25], with supervised ML models (random forest [RF], Gaussian naïve Bayes [GB], Bernoulli NB, and multinomial NB) and deep learning (artificial neural network [ANN]). To predict the importance of parameters under different classifications, an RF model was performed. The detailed information on R packages above was supplied as File S1.

### Metabolic analysis

Chemical standards including sodium danshensu, rosmarinic acid (RA), lithospermic acid A (LAA), LAB, dihydrotanshinone (DHDST), cryptotanshinone (CTSN), tanshinone I (TSN-I), and tanshinone IIA (TSN-IIA) were purchased from YUANYE Co., Ltd (Shanghai, China). All chemicals used in this study were of analytical or high-performance liquid chromatography (HPLC) grade.

All samples were dried at room temperature for some days until they reached a constant dry weight and milled to a homogeneous size that could be sieved through No. 100 mesh. Extraction and quantitation of active compounds were performed following a previously reported method [26]. Briefly, 0.1 mg of each sample was extracted in 10 ml of methanol under sonication for 30 min and the weight loss was supplemented with methanol after cooling. Then, 100 μl of the extract was evaporated with a rotovap at 35 °C, redissolved in 10 volumes, and centrifuged at 4 °C for 10 min. The supernatant was subjected to high-performance liquid chromatography-tandem triple quadrupole mass spectrometry (HPLC-MS/MS) analysis by an Agilent 1200-6410 LC/MS with the chromatographic column of Waters XSELECT CST-C18 (2.5 μm, 2.1 mm × 50 mm) at 35 °C, the flow rate was controlled at 0.3 ml/min, and the injection volume was 5 μl. The mobile phase was composed of acetonitrile (as phase A) and water containing 2 mmol/L ammonium acetate and 0.1% formic acid (phase B), and the elution method was as follows: 0 min (7% A)/2 min (7% A)/3 min (95% A)/8 min (95% A), and a final 6.5 min of equilibration post-run time. Quantitation was performed using multiple-reaction monitoring (MRM) mode; the MRM parameters are shown in Table S1. Data analysis was carried out by the Agilent Mass Hunter Workstation software. The quantitative results of each metabolite from each sample are listed in Table S2.

Mass spectrometry imaging (MSI) was carried out using the AP-SMALDI10 high-resolution matrix-assisted laser desorption/ionization tandem mass spectrometry (MALDI-MS) imaging ion source (TransMIT GmbH), which was operated at atmospheric pressure and coupled to a Q-Exactive Orbitrap mass spectrometer (Thermo Fisher). For root sections, a 2,5-dihydroxybenzoic acid matrix solution at a concentration of 30 mg∙ml^−1^ in acetone/water (0.1% trifluoroacetic acid) 1:1 v/v was treated first. All data were recorded on a 9.4-T MALDI FT-ICR (Fourier transform ion cyclotron resonance) MS equipped with a 355-nm Nd:YAG Smartbeam II laser (Bruker Daltonics, Billerica, MA). Mass spectra were acquired over the *m*/*z* range of 150 to 2,000 in both positive and negative ion modes with a mass resolution of 100,000 at *m*/*z* 400. For MSI experiments, tissue sections were analyzed with a spatial resolution of 75 μm, and a full-scan mass spectrum at 200 laser shots per pixel was acquired. Data were analyzed in Data Analysis version 4.0 and flexImaging software (version 4.1) (Bruker Daltonics, Billerica, MA). Metabolites were identified according to their mass spectrometry.

### Prediction of biomass and chemical contents using AlexNet

We utilized the AlexNet network to analyze images of *S. miltiorrhiza* roots. The feature extractor within AlexNet was responsible for extracting features from the input images. By applying convolutional and pooling layers, the information contained in the images was compressed into an *n*-dimensional feature vector containing all the relevant feature information of the input images. The predictor was employed to select and discard the extracted feature vector, ultimately condensing it into a prediction value. Subsequently, based on the discrepancy between the prediction value and the true labels, the network's weight parameters were continuously adjusted to progressively align the prediction value with the true labels.

## Results

### Workflow for the extraction and analysis of root phenotypic traits from imaging data

**Fig. 1.**
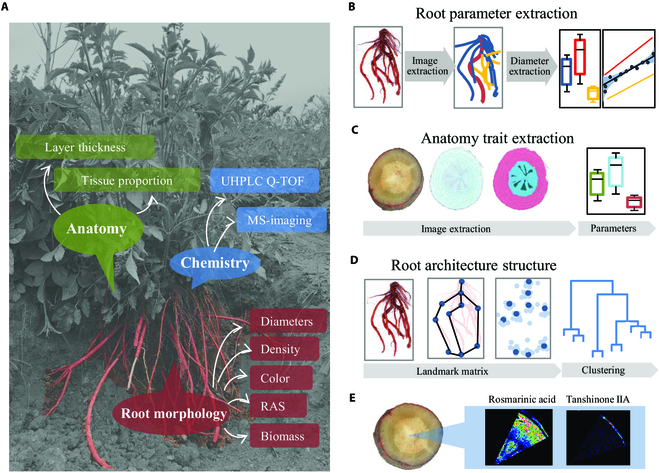
The workflow for the phenomics study of *S. miltiorrhiza* roots. (A) Map of trait categories included in this root phenotyping for *S. miltiorrhiza*. (B) Schematic presentations for image process, feature extraction, and analysis for agronomic traits using WinRHIZO and RhizoVision. (C) Schematic presentations for anatomy traits analyses using sections of *S. miltiorrhiza* roots. (D) Schematic presentations for modeling of root architecture structure using landmark method. (E) Schematic presentations for metabolic profiles on the section of *S. miltiorrhiza* roots using mass spectrometry imaging.

The phenotypic landscape of plant is highly complex and multidimensional [27]. In this study, adopting multiple root image analyzing tools, a framework for root phenotyping of *S. miltiorrhiza* was established at the levels of individual root branches and the entire root system, including agronomic features, anatomical traits, and RSA (Fig. 1A). WinRHIZO and RhizoVision Explorer, which both display morphology, topology, and architecture measurements from root images [22,28], were chosen to extract agronomic features from scanning graphics of *S. miltiorrhiza* roots (Fig. 1B). Rootscan, an imaging software codifying anatomical features of root cross-sections from microscope digital image [23], was employed to dissect the distribution of root tissues (Fig. 1C). Then, we used a landmark-based approach to describe the RSA of *S. miltiorrhiza*. The landmark model and morphospace were created by RootScape [24], and the corresponding *S. miltiorrhiza* roots were clustered into different RSA groups (Fig. 1D).

We also carried out the metabolic profiling of bioactive compounds in each root sample and their distributions in root tissues (Fig. 1E). Furthermore, we investigated the correlations between phenotypic traits and accumulation levels of metabolites in *S. miltiorrhiza* roots.

### Phenotyping of agronomic traits from scanning images of *S. miltiorrhiza* roots

**Fig. 2.**
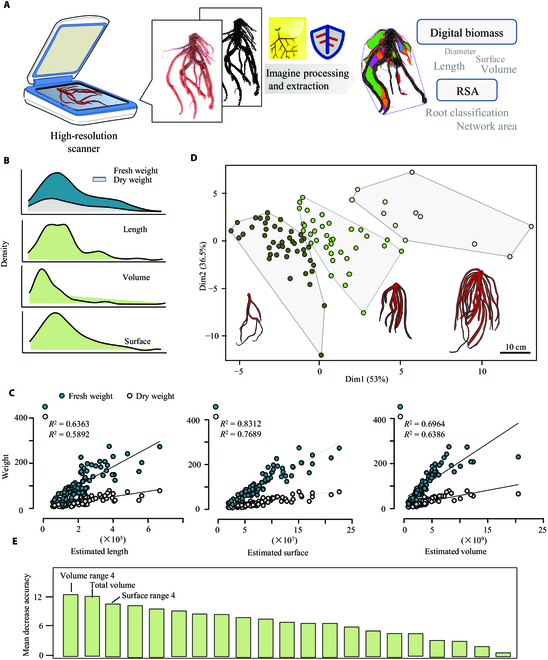
Phenotyping of *S. miltiorrhiza* root based on scanning images of the whole root system. (A) The workflow used for root phenotyping and data analysis. High-throughput imaging data from the WinRHIZO scanning system were imported and processed using WinRHIZO and RhizoVision. The extracted phenotypic traits were further evaluated for biomass estimation and RSA study. (B) The distribution of parameters related to the biomass of *S. miltiorrhiza* roots. (C) Linear regression analysis between real biomass (Fresh weight and Dry weight) and extracted parameters (Total Length, Total Surface, and Total Volume). (D) *K*-means clustering of *S. miltiorrhiza* roots samples (*n* = 92) according to the diameter ranges of each root branch. The root model represented the typical root structure among each cluster. The bar represented the size of each root model. (E) The importance evaluation of phenotypic traits according to the *K*-means clustering using the random forest model.

To capture agronomic traits, *S. miltiorrhiza* roots at the harvesting period were collected. Because the absorbing root is not used in the manufacturing of medicinal materials, it was discarded, and the main roots and lateral roots were kept for analyses. After washing, images of the whole root for each individual plant were obtained using a high-resolution scanner with both transmission and reflection modes. Using WinRHIZO and RhizoVision, a total of 81 parameters were obtained from 102 root images (Table S2). With consideration for the software's adaptability for *S. miltiorrhiza* roots and research objectives, we specifically selected parameters capable of characterizing biomass and root classes generated by RhizoVision (Fig. 2A and Table 1). In order to acquire parameters that reliably predict biomass, 3 parameters (Total Length, Total Surface Area, and Total Volume) were investigated. The normal distributions of these parameters were first analyzed separately (Fig. 2B). Consistently, the 3 parameters showed positive skewness distribution, whereas Total Surface displayed uniform distribution similar to that of the actual biomass (Fresh weight [FW] and Dry weight [DW]). We then examined the linear correlation between the actual biomass and digital biomasses (Fig. 2C). Both FW and DW exhibited a strong correlation with the predicted traits, with the highest correlation of Total Surface Area (*R*^2^ = 0.8312 for FW, and *R*^2^ = 0.7680 for DW), suggesting that the surface trait is a suitable image derived trait for estimation of root biomass.

**Table 1.** List of features extracted and selected for analysis of *S. miltiorrhiza* root images.

Software	Features extracted	Type	Description
RhizoVision/WinRHIZO	Total root length; average, median, and maximum diameter; volume; surface area; length; surface area; and volume histograms	Agronomic traits	The sum of the Euclidean distances between the connected skeletal pixels in the entire root topology
			The average, median, and maximum diameters that are computed across all root skeleton
			Volume and surface area are calculated as the sum of values from all skeletal pixels
			Length, surface area, and volume computed according to the diameter ranges
RootScan	Root cross-sectional area (RXSA)	Anatomical traits	All components of the cross-section
	Total cortical area (TCA)		All components inside RXSA but outside the stele
	Total stele area (TSA)		All areas of stele
	Xylem vessel area (VSA)		Total cross-sectional area of all metaxylem vessels
	Stele/cross-section		Proportion of cross-section occupied by stele
	Stele/cortex		Relative tissue allocation to stele and cortex
RootScape	Landmark placement defined by user	RSA	Defined landmarks that characterize the root system architecture

Besides, parameters produced by RhizoVision could be utilized to describe RSA [25]. Here, we conducted a root diameter classification analysis by selecting a range of root diameters. Based on the estimated diameter, each individual root branch was divided into 4 ranges as shown in Fig. S2 (Root length range 1 to 4, Surface area range 1 to 4, and Volume range 1 to 4), and their relative proportion in the whole root system was obtained additionally. The absorptive roots in range 1 were removed from statistical analysis. Applying the above data, all roots were clustered into 3 significantly distinguished groups using *K*-means clustering. As the root models showed, the roots in different sizes and biomass were grouped accordingly, providing a basis for the further study of their phenotypic or metabolic differences. Subsequently, based on the *K*-means clustering, RF was performed to figure out the most significant features (Fig. 2E). The mean decrease accuracy denotes the importance of the parameters or traits. Our result identified the Volume Range 4, Total Volume, and Surface Range 4 as the top 3 features, indicating their importance for the classification of *S. miltiorrhiza* roots.

### RSA construction using the landmark method

**Fig. 3.**
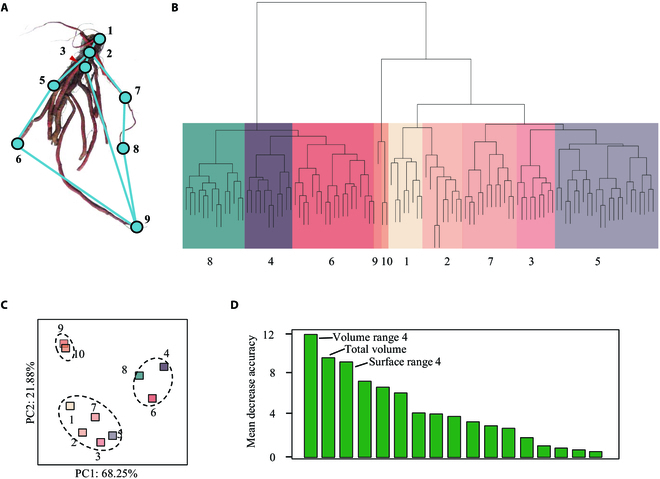
RSA analyses of *S. miltiorrhiza* roots by the landmark method. (A) Landmark placement for *S. miltiorrhiza* root. We defined a set of 9 landmarks covering the overall outline of the root system and primary roots. (B) Samples of *S. miltiorrhiza* root were grouped into 10 clades according to the landmark placement. The branch of each group was labeled in different colors. The clades were then clustered into 3 categories by PCA (C). (D) The importance evaluation of phenotypic traits according to the landmark clustering (10 clades) using the random forest model.

As described above, the root classification can characterize RSA in terms of morphology (Fig. 2D). However, RSA can be defined in various ways, such as morphology, topology, distribution, and architecture to address the diverse physiological concerns of plant roots [7]. RootScape, a landmark-based technique, was employed for comprehensive RSA description [24]. A 9-point landmark model was created to capture the RSA of *S. miltiorrhiza*, describing the distances between landmarks that were defined by the primary root (points 1 and 2), the outermost lateral roots (points 2, 3, 5, 6, 7, and 8), and the depth of the root system (point 9) (Fig. 3A). Following the same principle, each root was assigned with labels, and then a distance matrix between each pair of landmarks was generated (Table S3). As a result, the root samples were clustered into 10 groups (Fig. 3B), which generally belonged to 3 categories (Fig. 3C). Based on such classification, we further identified key features using RF modeling, which again represented Volume Range 4, Total volume, and Surface Range 4 as the most important traits.

### Classification evaluation using ML algorithms

ML algorithms are expected to provide more accurate classification of plant roots according to RSA features [25]. We conducted tests with various ML algorithms to identify the most suitable model, including unsupervised algorithm, supervised algorithm (RF, GB, Bernoulli NB, and multinomial NB), and deep learning model (ANN). Based on the clustering result of *K*-means (all samples in 3 clusters, Fig. 2D) and the landmark method (all samples in 10 clusters, Fig. 3B), the prediction accuracy for each ML model was evaluated (Table 2). As a result, *K*-meaning clustering showed high accuracy at 1.00 with the accuracy coefficient of 2 supervised algorithms (RF and GB). However, when assessed using all algorithms, the accuracy according to the landmark categories was poor. These findings indicate that RF and GB are the most effective models for *S. miltiorrhiza* root classification, and *K*-means clustering was superior to the landmark method using the current landmark placements.

**Table 2.** Prediction of accuracy evaluations for different machine learning models.

Model	Unsupervised	Supervised				Artificial neural network
	*K*-means	Random forest	Gaussian naïve Bayes	Bernoulli NB	Multinomial NB	
*K*-means clustering	0.765	1.000	1.000	0.655	0.483	0.241
Landmark model	0.129	0.161	0.129	0.194	0.129	0.194

### Correlation between bioactive metabolites and phenotypic traits

**Fig. 4.**
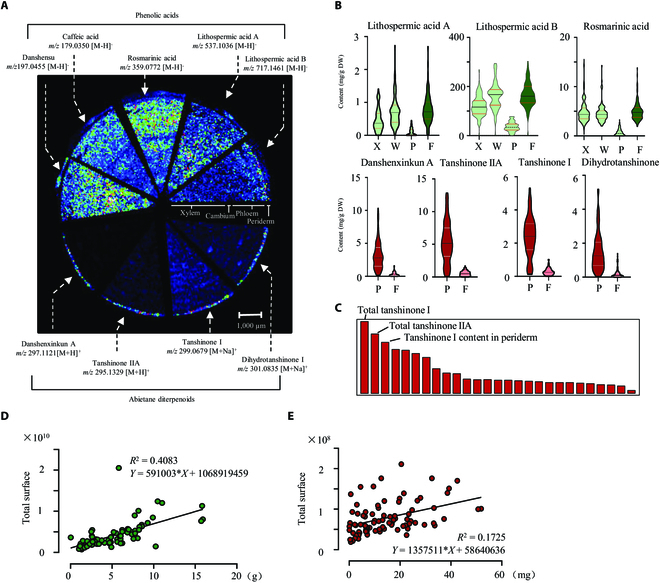
Metabolic profiling of pharmaceutic metabolites in *S. miltiorrhiza* roots and their correlation with phenotypic traits. (A) Mass spectrometry imaging by MALDI-MS showed the spatial distribution of 2 classes of effective metabolites on a section of *S. miltiorrhiza* root with a spatial resolution of 75 μm. The ion strength is color-coded (white = maximal signal and black = minimal signal) and normalized. (B) Content of phenolic acids and tanshinones in different root tissues measured by HPLC-MS/MS. X, xylem; W, phloem and cambium layers; P, periderm, F, the whole root segment. (C) ML represented the most variable metabolic traits according to *K*-means clustering shown in Fig. 2E. The top 3 parameters were labeled. Linear regression analysis showed that the production of LAB (D) was significantly correlated to the selected phenotypic trait (Total Surface), while tanshinone IIA (E) was small but significantly correlated to the selected phenotypic trait (Total Surface).

Phenolic acids and tanshinones are the primary active components of *S. miltiorrhiza*. Among them, danshensu, LAB, and tanshinone are synthesized in specific *S. miltiorrhiza* species or organs [16]. To understand the correlation between bioactive metabolites and phenotypic traits, MSI was conducted to profile the distribution of these metabolites in the root. Using an AP (atmospheric pressure)-MALDI MS imaging system, a total of 12 bioactive metabolites were identified. Phenolic acids such as danshensu (*m/z* 197.0455, [M-H]^−^), caffeic acid (*m/z* 179.0350, [M-H]^−^), RA (*m/z* 359.0772, [M-H]^−^), LAA (*m/z* 537.1036, [M-H]^−^), and LAB (*m/z* 717.1461, [M-H]^−^) were identified according to their accurate mass in negative mode. The image of the *S. miltiorrhiza* root sections showed that they were distributed in almost all root tissues. In positive mode, tanshinone metabolites as Danshenxinkun A (*m/z* 297.1121, [M+H]^+^), TSN-IIA (*m/z* 295.1329, [M+H]^+^), TSN-I (*m/z* 299.0679, [M+Na]^+^), and DHDST I (*m/z* 301.0835, [M+Na]^+^) were identified, which were only or highly accumulated in the periderm layer (Fig. 4A). Subsequently, we performed accurate quantification of 7 major active metabolites in different root tissues using the HPLC-MS/MS - method (Fig. 4B and Table S4). Since the cambium cells could not be individually distinguished and separated, this tissue was examined together with the phloem layers. Consequently, the root tissues were manually separated into 3 parts, periderm, phloem, and xylem. Consistent with the MS-imaging results, phenolic acids (LAA, LAB, and RA) were relatively highly accumulated in the inner tissues (phloem, cambium, and xylem), and had a very low content in the periderm. On the contrary, TAs (Danshenxinkun A, TSN-IIA, TSN-I, and DHDST) were only detected in the periderm (Fig. 4B). Besides the separated tissues, the content of each metabolite in the whole root segments was also detected, and they were all similar to that of inner tissues. Given the different distributions of these 2 classes of metabolites in the root, we infer that the TA content of *S. miltiorrhiza* roots may be associated with the phenotypic characteristics of the periderm. The correlation between metabolites and phenotypic traits was then established using Pearson correlation analysis (Table S5). According to the correlation network, there were substantial associations between the accumulation level of CTSN or LAB and several phenotypic features, including Volume Range 4, Length Range 4, and Total Surface. These results indicate that the production of these metabolites is probably determined by the roots in range 4, the main root parts of the *S. miltiorrhiza* root system. RF was also performed to figure out the most significant metabolic traits that were influenced by phenotypic features (Fig. 4C). However, yields and content of tanshinones, not phenolic acids, seemed to be the most important metabolic traits based on the *K*-means classification (Fig. 2D), probably due to the different accumulation patterns between tanshinones and phenolic acids.

Owing to the correlation between accumulation patterns of metabolites and phenotypic features, we hypothesized that the phenotypic features could be used to characterize the production of bioactive compounds in roots of *S. miltiorrhiza* population. For this purpose, we examined the linear regression relationship between metabolite contents and biomass traits. Because the Total Surface had the highest correlation to actual biomass, it was chosen for analysis. As a result, among all the tested metabolites, the production of LAB (*R*^2^ = 0.4083 and *P* < 0.0001) and TSN-IIA (*R*^2^ = 0.1725 and *P* < 0.0001) in root was well correlated with their digital biomass based on Total Surface (Fig. 4D and E), implying that the content of these bioactive chemicals could be measured according to their digital biomass without quantitative detection using the traditional chemical method.

## Discussion

The aim of this study was to establish a workflow for characterizing the phenotypic traits of *S. miltiorrhiza* roots. The phenotypic data were further used for valid biomass estimation, root classification, and prediction of bioactive metabolites in the root.

Different from previous root phenotyping studies, we tried to conduct a multi-dimensional characterization of *S. miltiorrhiza* root, which requires different analyzing platforms for various categories [29]. Apparently, there is no readily available root phenotyping tool to meet these requirements simultaneously. Therefore, we chose different ready-made root analysis tools to extract phenotypic traits in different categories (morphology, architecture, anatomy, and chemistry), which were further combined together. Since these tools were not specially designed for *S. miltiorrhiza* roots, some particular traits were not utilized here. For example, root tips can be recognized and counted by RhizoVision or WinRhizo. Such information is meaningful for small root systems in *Arabidopsis* and wheat [30]; however, it may have no impact on the biomass estimation of mature *S. miltiorrhiza*. For anatomical traits, we chose RootScan, which was designed for maize [23], to obtain the proportion of different root tissues in the primary root. However, RootScan is unable to collect key characteristics for which we are more concerned, such as the proportion of periderm. Thus, the above disadvantages of using nonspecific root image analysis software indicate that it is necessary to further establish a species-specific phenotypic analysis software for *S. miltiorrhiza*.

RSA is a complex concept and can be described from different perspectives. There is still no simple definition to distinguish the key features of RSA. Most research on RSA is based on overall parameters through root scanning [25] or 3-dimensional modeling [31,32]. Here, we suggest that the landmark method can potentially simplify the description of RSA. Because landmark topology can be adapted to different plant species and organs, the investigators can define placement according to a certain purpose. Landmark topology has been successfully employed to evaluate the morphology of floral organs, such as style and petal [33,34]. However, it is not frequently used in root phenotyping [24], which is probably hindered by root imaging technology. In this work, we created a 9-landmark model mainly to characterize the primary root and the skeleton of *S. miltiorrhiza* roots. Although the accuracy assessment by ML algorithms did not provide strong support for the landmark-mediated roots classification, it does not mean that the landmark method is not suitable for studies on RSA, which might require a different or more detailed set of landmarks.

The main goal of root phenotyping in current studies is to quantify morphological and architectural root traits for root modeling, biomass measurement, trait correlation, and dynamic changes of morphological traits [35]. In this study, we successfully predicted the biomass of *S. miltiorrhiza* roots according to their phenotypic traits. We also tried to establish a prediction model for metabolite contents in roots using phenotypic traits, revealing that the production of metabolites could be estimated according to the biomass-related parameters such as Total Surface (Fig. 4D and E). Several recent studies have shown potential applications for the estimation of valuable metabolites using high-throughput image analyses. For example, Liu et al. [36] developed a visual discrimination model of buckwheat flavonoid content based on color code values (CMYK) of seed images by a neural network (AlexNet). Interestingly, the red color of *S. miltiorrhiza* roots is due to the accumulation of tanshinones, thus indicating the possibility of predicting tanshinone content by root color using computer vision technologies. However, although phenotypic traits can efficiently reflect the contents of metabolites, the accumulation of these metabolites is also tightly associated with their specific physiological functions and varies under different conditions. Here, we only discussed the relationship between phenotypic traits and tanshinone contents. In reality, phenotypic studies can also address developmental processes, stress resistance, and other physiological phenomena. If we can integrate these diverse physiological aspects through phenotypic profiling studies, it may be possible to elucidate the precise physiological functions of tanshinones and explore intriguing questions related to their specific accumulation in the periderm and other physiological significance.

Currently, most image-based root analysis software relies on semi-automatic or manual graphic analyses, which is not effective for situations with large sample sizes. Deep learning of plant root images based on neural network algorithms is expected to be highly useful for high-throughput plant phenotyping [37]. Here, AlexNet was performed to estimate the biomass of *S. miltiorrhiza*. The dataset used in this experiment consists of a total of 91 images of *S. miltiorrhiza* roots. After random partitioning and data augmentation, the training set contained 540 images (including 54 original images expanded through geometric transformations such as rotation and translation). The validation set contains 180 images, which was expanded in the same way as the training set. The test set contains nineteen images. The AlexNet network structure is shown in Fig. S3A. After 200 rounds of training, we conducted testing on the test set. As a result, it was found that the predicted biomass closely approximated the average fresh weight, albeit with a significant discrepancy (51.016 g on average) from the actual fresh weight (Fig. S3B). Likewise, the predicted dry weight failed to accurately reflect the true dry weight with a discrepancy of 18.134 g in average and exhibited a larger deviation from the average (Fig. S3C). It can be observed that the biomass predictions based on neural networks tend to stabilize, rather than accurately reflecting the true differences in biomass between samples. This may be due to the limited number of samples in the training set.

Altogether, we established a framework for phenotyping of *S. miltiorrhiza* roots, focused on the phenotyping of mature roots and searching for the correlations between phenotypic traits and bioactive metabolites. In fact, the framework is expected to be used in more extensive research on the root physiology of *S. miltiorrhiza*. For example, the parameters correlated to biomass can be used to build dynamic models of root development during the life cycle [4], and root responses to biotic or abiotic stress [38], or root variations in response to different nutrition conditions and environments [39]. According to the growth modeling and variation of key parameters, breeders and farmers may obtain useful information for the fine cultivation of this herb. On the other hand, despite the publication of several versions of the *S. miltiorrhiza* genome [40–42], there are still no cultivated varieties of *S. miltiorrhiza* with definite genotypes, leading to the lagging of *S. miltiorrhiza* breeding. The phenotyping and classification approach we used here may aid in the correct identification of *S. miltiorrhiza* varieties and the selection of critical traits, thus providing a new point for *S. miltiorrhiza* breeding.

## Data Availability

The data and code that support the findings of this study are available on Zenodo doi: 10.5281/zenodo.8151629.

## Supplementary Materials

Supplementary 1 Table S1. MRM parameters. Table S2. Phenotypic traits captured in this study using multiple software. Table S3. Landmark matrix produced by RootScape according to the 9-landmark set as described in Fig. 3A. Table S4. Metabolic profiling of bioactive metabolites in different root tissues measured by LC-QQQ-MS. Table S5. Pearson correlation analysis of metabolic and phenotypic traits. Fig. S1. Workflow for anatomy trait analyses. Fig. S2. Diameter ranging analyses of *S. miltiorrhiza* root using RhizoVision. Fig. S3. Prediction of root biomass using AlexNet. File S1. Packages for ML algorithms used in this study.
