# Supplementary material for: Phenotyping of Salvia miltiorrhiza Roots Reveals Associations between Root Traits and Bioactive Components
Source: Plant Phenomics. 2023 Oct 2;5:0098. doi: 10.34133/plantphenomics.0098 (PMC10545446; doi:10.34133/plantphenomics.0098)
Supplement: Supplementary 1 — Table S1. MRM parameters. Table S2. Phenotypic traits captured in this study using multiple software. Table S3. Landmark matrix produced by RootScape according to the 9-landmark set as described in Fig. 3A. Table S4. Metabolic profiling of bioactive metabolites in different root tissues measured by LC-QQQ-MS. Table S5. Pearson correlation analysis of metabolic and phenotypic traits. Fig. S1. Workflow for anatomy trait analyses. Fig. S2. Diameter ranging analyses of S. miltiorrhiza root using RhizoVision. Fig. S3. Prediction of root biomass using AlexNet. File S1. Packages for ML algorithms used in this study. [file plantphenomics.0098.f1.zip › File S1.docx]

**Supplementary data**

**File S1** Packages for ML algorithms used in this study.
