# Supplementary material for: Phenotyping of Salvia miltiorrhiza Roots Reveals Associations between Root Traits and Bioactive Components
Source: Plant Phenomics. 2023 Oct 2;5:0098. doi: 10.34133/plantphenomics.0098 (PMC10545446; doi:10.34133/plantphenomics.0098)
Supplement: Supplementary 1 — Table S1. MRM parameters. Table S2. Phenotypic traits captured in this study using multiple software. Table S3. Landmark matrix produced by RootScape according to the 9-landmark set as described in Fig. 3A. Table S4. Metabolic profiling of bioactive metabolites in different root tissues measured by LC-QQQ-MS. Table S5. Pearson correlation analysis of metabolic and phenotypic traits. Fig. S1. Workflow for anatomy trait analyses. Fig. S2. Diameter ranging analyses of S. miltiorrhiza root using RhizoVision. Fig. S3. Prediction of root biomass using AlexNet. File S1. Packages for ML algorithms used in this study. [file plantphenomics.0098.f1.zip › renamed_eb970.docx]

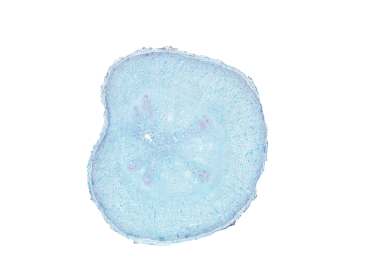

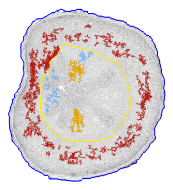

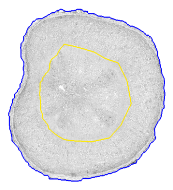

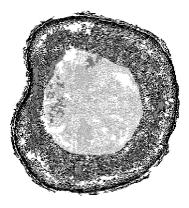


Cross section

Cortex

Xylem

Vessels

Imagine processing and extraction by RootScan

Stele

Tissues Area/Ratio

Stele

Cortex

Xylem

\

**Figure S1 Workflow for anatomy traits analyses.**

The images of root section were analyzed by RootScan (Burton et al., 2012). Tissues including stele, cortex, and xylem were identified semi-manually for their area estimation.


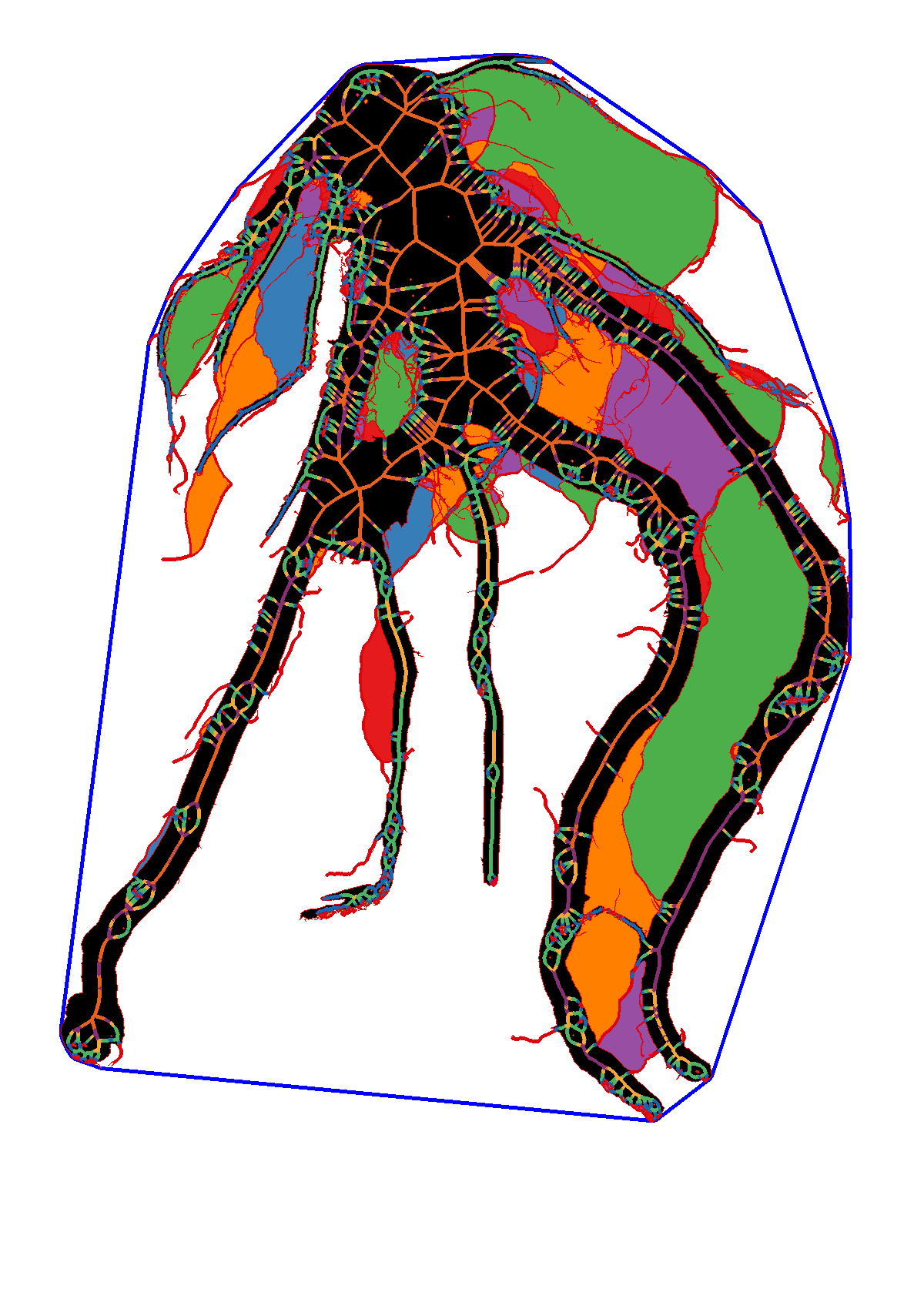


**Figure S2 Diameter ranging analyses of *S. miltiorrhiza* root using RhizoVisio**n.

The root branches were distinguished according to their estimated diameter values measured by RhizoVision. The skeleton for root branches in different diameter ranges were represented in different colors. Range 1 (0-20.00 mm, red), range 2 (20.00-40.00 mm, blue), range 3 (40.00-60.00 mm, green), and range 4 (above 60.00 mm, orange).


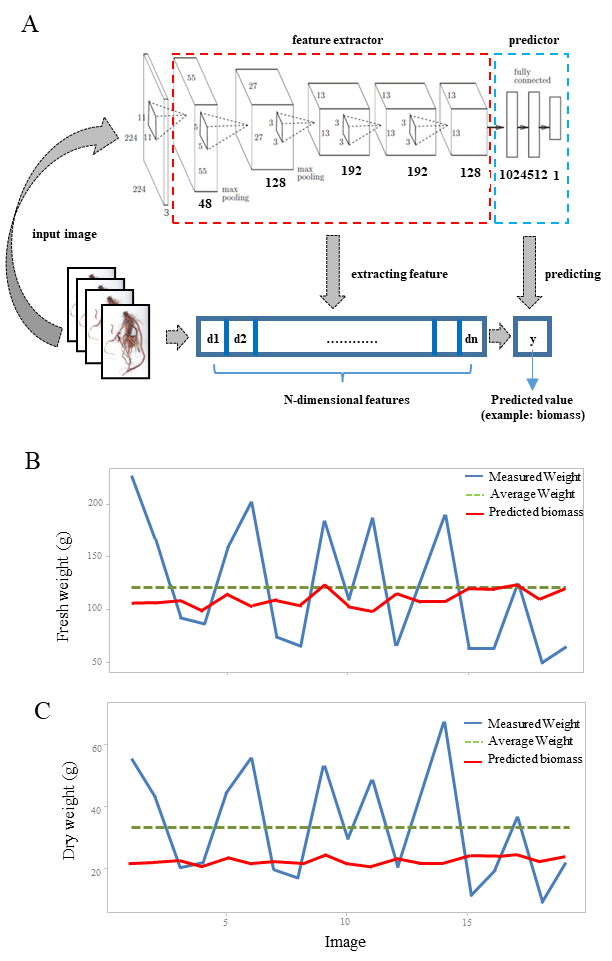


**Figure S3 Prediction of root biomass using AlexNet.** (A) Structure of AlexNet. (B, C) Comparation of predicted biomass and measured biomass (fresh and dry weight).
